# Involvement of Histamine and RhoA/ROCK in Penicillin Immediate Hypersensitivity Reactions

**DOI:** 10.1038/srep33192

**Published:** 2016-09-13

**Authors:** Jiayin Han, Yan Yi, Chunying Li, Yushi Zhang, Lianmei Wang, Yong Zhao, Chen Pan, Aihua Liang

**Affiliations:** 1 Institute of Chinese Materia Medica, China Academy of Chinese Medical Sciences, Dongcheng District, Beijing 100700, China

## Abstract

The mechanism of penicillin immediate hypersensitivity reactions has not been completely elucidated. These reactions are generally considered to be mediated by IgE, but penicillin-specific IgE could not be detected in most cases. This study demonstrated that penicillin was able to cause vascular hyperpermeability in a mouse model mimicking clinical symptoms of penicillin immediate hypersensitivity reactions. The first exposure to penicillin also induced immediate edema and exudative reactions in ears and lungs of mice in a dose-dependent manner. Vasodilation was noted in microvessels in ears. These reactions were unlikely to be immune-mediated reactions, because no penicillin-specific IgE was produced. Furthermore, penicillin treatment directly elicited rapid histamine release. Penicillin also led to F-actin reorganization in human umbilical vein endothelial cells and increased the permeability of the endothelial monolayer. Activation of the RhoA/ROCK signaling pathway was observed in ears and lungs of mice and in endothelial cells after treatment with penicillin. Both an anti-histamine agent and a ROCK inhibitor attenuated penicillin immediate hypersensitivity reactions in mice. This study presents a novel mechanism of penicillin immediate hypersensitivity reactions and suggests a potential preventive approach against these reactions.

Penicillin causes immunoglobulin E (IgE)-mediated immediate allergic hypersensitivity reactions (AHRs) with serious consequences such as urticaria, pruritus, angioedema, bronchospasm, and anaphylaxis. Approximately 10% of the population claim to have some type of penicillin allergy; however, less than 10% of them are confirmed to be truly allergic^1^,^2^,^3^. Most individuals who claim to have a history of penicillin allergy do not have detectable specific IgE, and lack of positive results from skin testing and oral challenge^4^,^5^,^6^,^7^. It has been suggested that some β-lactam hypersensitivity-related symptoms are generally not immunologic reactions in nature^8^ and are frequently dose related^9^. A rapid release of histamine and other inflammatory mediators can also be induced by a non-immunologic pathway and can lead to non-allergic hypersensitivity reactions (NAHRs)^10^,^11^,^12^,^13^,^14^. NAHRs mimic IgE-mediated AHRs with identical clinical symptoms, including angioedema, urticaria, bronchospasm, gastrointestinal signs, and anaphylaxis^15^. Because of the clinical similarities between IgE-mediated reactions and non-allergic immediate hypersensitivity reactions, it is very difficult to distinguish between them^16^. Many adverse reactions to drugs are not immunologically mediated but are recorded as an “allergy”^17^. Whether penicillin induces NAHRs in addition to immediate AHRs is unknown.

The small GTPase RhoA and its downstream effector, Rho-associated kinase (ROCK), play an essential role in regulating a number of cellular processes, including cell motility, proliferation, and permeability^18^,^19^,^20^,^21^. RhoA acts as a molecular switch by alternating between an active GTP-bound state and an inactive GDP-bound state. ROCK controls actomyosin contractility via direct phosphorylation of the myosin light chain (MLC) and suppression of myosin phosphatase^19^,^22^. Previous studies have shown that activation of the RhoA/ROCK signaling pathway mediates endothelial hyperpermeability in response to various chemicals or mediators, such as thrombin^23^, histamine^24^,^25^, vascular endothelial growth factor^26^, cytokines^27^, high glucose^28^ and lipopolysaccharides^29^. Because clinical signs of penicillin immediate hypersensitivity reactions including angioedema, urticaria, bronchospasm, and anaphylaxis are closely related to endothelial hyperpermeability, we hypothesize that the RhoA/ROCK signaling pathway is involved in penicillin hypersensitivity reactions.

The mechanism of penicillin immediate hypersensitivity reactions has not been completely elucidated. This study is the first to investigate whether penicillin treatment might induce IgE-independent vascular hyperpermeability in a mouse model which mimics the typical clinical symptoms of immediate allergic reactions to penicillin. We demonstrate that histamine release and the RhoA/ROCK signaling pathway play important roles in penicillin NAHRs, and the results suggest that an anti-histamine agent and a ROCK inhibitor might be useful for the prevention and treatment of some penicillin hypersensitivity reactions.

## Results

### Penicillin Induces Immediate Non-allergic Hypersensitivity Reactions in Mice

**Figure 1:**
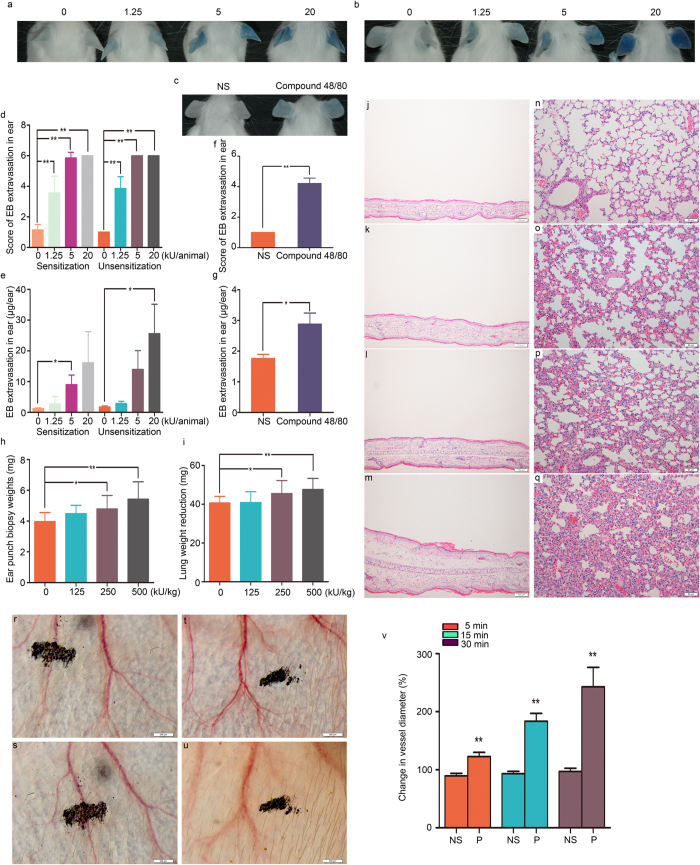
Vascular leakage and pathological changes in the ears and lungs of mice. (**a–c**) Vascular leakage in the sensitized mice received challenge treatment of penicillin/EB, unsensitized mice received a single dose of penicillin/EB, and naive mice received a single dose of compound 48/80/EB. (**d,e**) EB extravasation for the ears of the sensitized mice received challenge treatment with penicillin/EB or unsensitized mice received a single dose of penicillin/EB (n = 7 per group). (**f,g**) EB extravasation for the ears of mice received a single dose of compound 48/80/EB (n = 7 per group). (**h**) Weights of ear punch biopsy (n = 10 per group). (**i**) The differences in weight between wet and dry lungs (n = 10 per group). (**j–m**) Microscopic examination of ears ((**j**) normal saline; (**k**) 125 kU/kg penicillin; (**l**) 250 kU/kg penicillin; (**m**) 500 kU/kg penicillin). (**n–q**) Microscopic examination of lungs ((**n**) normal saline; (**o**) 125 kU/kg penicillin; (**p**) 250 kU/kg penicillin; (**q**) 500 kU/kg penicillin). (**r–u**) Vascular change of ear ((**r**,**s**) pre-dose and 30 minutes after normal saline dosing; (**t,u**) pre-dose and 30 minutes after 500 kU/kg penicillin dosing). (**v**) Change in vessel diameter (n = 6 per group). *p < 0.05, **p < 0.01 compared with the normal saline-treated group; one-way ANOVA or Kruskal-Wallis H test. Error bars represent the s.e.m.

To examine the extent of vascular leakage induced by penicillin, we used Evans blue (EB) as a marker of plasma protein extravasation, because it binds plasma albumin with high affinity and forms an albumin-EB complex. This property has been widely applied to assess vascular leakage and the extent of inflammatory extravasation^30^,^31^. We injected penicillin intravenously with EB and determined vascular leakage by observing EB leakage in the ear. As a control, EB alone did not cause visible vascular leakage in mice, as indicated by EB extravasation. Dose-dependent vascular leakage was observed in ears after penicillin-sensitized mice received a challenge treatment of penicillin (1.25, 5, or 20 kU/mouse) in combination with EB. EB extravasation occurred at approximately 7–10 minutes and reached a peak at approximately 20 minutes after penicillin treatment. Interestingly, marked dose-dependent EB extravasation was also observed in unsensitized mice receiving a single dose of penicillin/EB at the same dose levels as those for the penicillin challenge in sensitized mice. Prior sensitization did not enhance the extent of penicillin-induced EB extravasation compared with that in the penicillin single-dose group (Fig. 1a,b,d,e). The microvascular leakage of EB noted in penicillin-treated mice was similar to that caused by compound 48/80, a mast cell degranulation inducer (Fig. 1c,f,g).

We further ruled out the role of penicillin-specific IgE in vascular leakage. The passive cutaneous anaphylaxis (PCA) test is a standard method of measuring allergen-specific IgE antibody levels in allergy mouse models^32^. As a positive control, ovalbumin (OVA) induced a 100% positive response, whereas no visible reaction was observed in penicillin-treated groups (Table 1). This result indicated that penicillin-specific IgE was not responsible for the vascular hyperpermeability in the mice treated with penicillin.

To additionally investigate the IgE-independent reactions induced by penicillin in mice, ear and lung weight data were obtained at 30 minutes after penicillin treatment, and histological observations were performed to evaluate whether penicillin causes ear edema and pulmonary exudation. A single dose of penicillin at 250 or 500 kU/kg significantly enhanced ear weight by 20% and 35% compared with that in the control group (Fig. 1h). The increase in ear weight was dose dependent. The microscopic examination confirmed that the increase in ear weight in the penicillin-treated groups was due to edema, which was characterized by ear thickening, inflammatory cell infiltration, and microvascular dilation (Fig. 1j–m). Moreover, a single dose of penicillin at 250 or 500 kU/kg also resulted in pulmonary inflammation. The differences in weight between wet and dry lungs (lung weight reduction) increased by 12% and 17%, respectively, compared with that of the control group (Fig. 1i). The differences in weight were also dose dependent. The histological changes, including congestion, edema with alveolar septal broadening, and perivascular and peribronchial monocyte infiltration were observed in the lungs of mice in the penicillin-treated groups (Fig. 1n–q).

We next directly observed the local vascular changes in ears. Treatment with 500 kU/kg penicillin significantly dilated microvessels. Vascular dilation, edema and leakage were noted ever since 5 minutes and got severe gradually within 30 minutes after penicillin treatment (Fig. 1r–u). The vessel diameter change of penicillin group was approximately 1.4-fold, 2-fold, and 2.5-fold compared with normal saline group at 5 minutes, 15 minutes and 30 minutes after treatment (Fig. 1v).

### Histamine Release Contributes to Penicillin NAHRs

**Figure 2:**
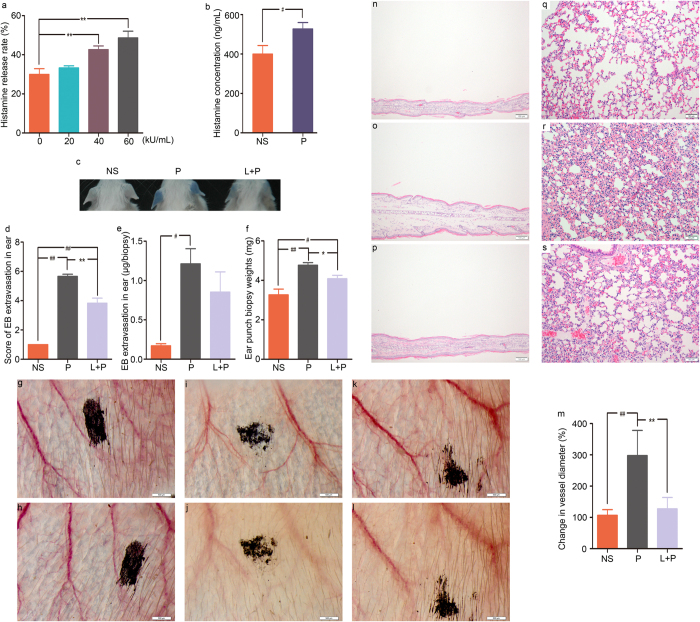
Penicillin-induced histamine release and the inhibitory effect of loratadine on penicillin NAHRs. (**a**) Histamine release in RBL-2H3 cells treated with penicillin (n = 12 per group). (**b**) Plasma histamine concentration of mice received a single dose of penicillin (n = 6 per group). (**c**) Vascular leakage in mice received penicillin/EB with or without pretreatment with loratadine. (**d–f**) EB extravasation for ears and weights of ear punch biopsy for mice received penicillin/EB with or without pretreatment with loratadine (n = 6 per group). (**g–l**) Vascular change of ear ((**g,h**) pre-dose and 30 minutes after normal saline dosing; (**i,j**) pre-dose and 30 minutes after penicillin dosing; (**k,l**) pretreatment with loratadine, and pre-dose and 30 minutes after penicillin dosing). (**m**) Change in vessel diameter (n = 6 per group). (**n–p**) Microscopic examination of ears ((**n**) normal saline; (**o**) 500 kU/kg penicillin; (**p**) 3 mg/kg loratadine + 500 kU/kg penicillin). (**q–s**) Microscopic examination of lungs ((**q**) normal saline; (**r**) 500 kU/kg penicillin; (**s**) 3 mg/kg loratadine + 500 kU/kg penicillin). NS: normal saline; P: 500 kU/kg penicillin; L + P: 3 mg/kg loratadine + 500 kU/kg penicillin. *p < 0.05, **p < 0.01 compared with the penicillin-treated group; ^#^*p* < 0.05, ^##^*p* < 0.01 compared with the normal saline-treated group; one-way ANOVA or Kruskal-Wallis H test. Error bars represent the s.e.m.

We tested whether penicillin directly elicits histamine release by performing *in vitro* and *in vivo* experiments. In the *in vitro* experiment, histamine release in rat basophilic leukemia-2H3 (RBL-2H3) cells markedly increased in a concentration-dependent manner after the cells were treated with penicillin (20, 40, or 60 kU/mL) for 30 minutes (Fig. 2a). In the *in vivo* experiment, the histamine concentration in plasma was notably higher in mice treated with a single dose of 500 kU/kg penicillin, with a 33% higher concentration than that in the control group (Fig. 2b). Moreover, pretreatment with the anti-histamine drug loratadine significantly alleviated the vascular leakage, ear swelling and pulmonary inflammation induced by a single dose of 500 kU/kg penicillin. Penicillin-induced vascular hyperpermeability, increased ear weight, vasodilation and edema were ameliorated in the loratadine pretreatment group (Fig. 2c–m). Microscopic examination showed that penicillin-induced edema, congestion and inflammatory cell infiltration in the ears and lungs were attenuated by pretreatment with loratadine (Fig. 2n–s).

### Penicillin Enhances Endothelial Monolayer Permeability through Activation of the RhoA/ROCK Signaling Pathway

**Figure 3:**
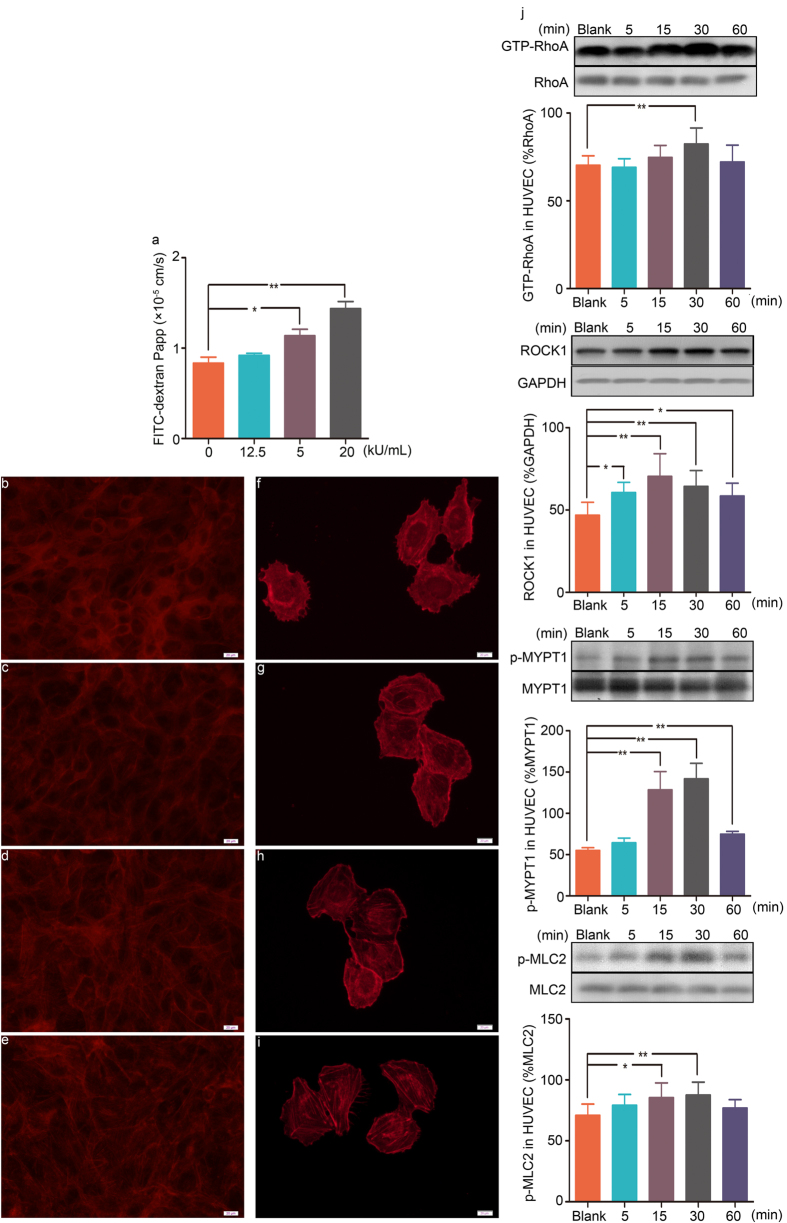
The effect of penicillin on endothelial monolayer permeability, F-actin distribution and GTP-RhoA, ROCK1, p-MYPT1 and p-MLC2 expression in HUVECs. (**a**) Fluorescence apparent permeability coefficients of FITC-dextran through the HUVEC monolayer (n = 3, per group). (**b–i**) F-actin formation and assembly in confluent endothelial monolayer or un-confluent HUVECs ((**b,f**) untreated cells; (**c,g**) 1.25 kU/mL penicillin; (**d,h**) 5 kU/mL penicillin; (**e,i**) 20 kU/mL penicillin). (**j**) GTP-RhoA, ROCK1, p-MYPT1 and p-MLC2 expression in HUVECs incubated with 5 kU/mL penicillin for different time courses (n = 6, per group). Blank: untreated cells. *p < 0.05, **p < 0.01 compared with untreated cells; one-way ANOVA. Error bars represent the s.e.m.

Because penicillin-induced vascular leakage is correlated with vascular endothelial barrier dysfunction, we evaluated whether penicillin enhances endothelial permeability through an *in vitro* experiment. By assessing FITC-dextran diffusion through the confluent endothelial monolayer, we found that incubation with penicillin (1.25, 5, or 20 kU/mL) for 1 hour significantly increased the human umbilical vein endothelial cell (HUVEC) monolayer permeability (characterized by fluorescence apparent permeability coefficients, P_app_) in a concentration-dependent manner (Fig. 3a). These results demonstrated that penicillin enhanced endothelial permeability, which in turn led to vascular hyperpermeability.

To determine whether penicillin induces alterations in the actin cytoskeleton, actin filaments (F-actin) were stained and observed. Rhodamine-phalloidin staining of confluent endothelial monolayer or un-confluent HUVECs both demonstrated that stimulation with penicillin (1.25, 5, or 20 kU/mL) for 1 hour resulted in noticeable F-actin accumulation and reorganization in HUVECs. F-actin intensity was increased in a concentration-dependent manner (Fig. 3b–i). Stress fiber formation and assembly may dissociate cell connections and cause a separation of cells and looseness of the endothelial barrier, thereby contributing to an increase in endothelial permeability.

We further determined whether the RhoA/ROCK signaling pathway is involved in the penicillin-induced endothelial permeability increase and F-actin reorganization in HUVECs. The results indicated that incubation with 5 kU/mL penicillin activated the RhoA/ROCK signaling pathway in a time-dependent manner. The protein levels of GTP-bound RhoA (GTP-RhoA), ROCK1, phospho-myosin phosphatase targeting subunit 1 (p-MYPT1) and phospho-myosin light chain 2 (p-MLC2) in HUVECs were increased within 15 minutes of penicillin exposure and reached maximum levels after 30 minutes of penicillin treatment (Fig. 3j).

### Fasudil Inhibits Penicillin-induced Endothelial Barrier Dysfunction

**Figure 4:**
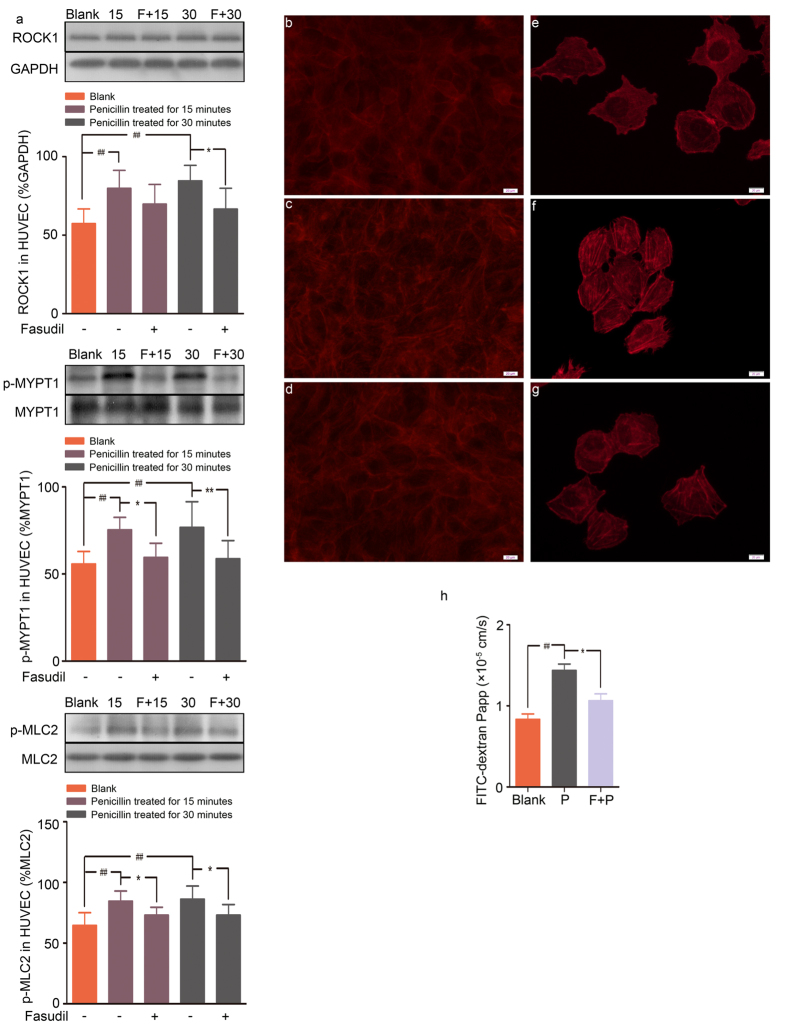
The inhibitory effect of fasudil hydrochloride on penicillin-induced upregulation of ROCK1, p-MYPT1 and p-MLC2 expression, F-actin reorganization and endothelial monolayer hyperpermeability in HUVECs. (**a**) ROCK1, p-MYPT1 and p-MLC2 expression in HUVECs incubated with 5 kU/mL penicillin with or without pretreatment with 10 μM fasudil hydrochloride (n = 6, per group). (**b–g**) F-actin formation and assembly in confluent endothelial monolayer or un-confluent HUVECs ((**b,e**) untreated cells; (**c,f**) 20 kU/mL penicillin; (**d,g**) 10 μM fasudil hydrochloride +20 kU/mL penicillin). (**h**) Fluorescence apparent permeability coefficients of FITC-dextran through the HUVEC monolayer (n = 3, per group). Blank: untreated cells; P: 20 kU/mL penicillin; F + P: 10 μM fasudil hydrochloride +20 kU/mL penicillin. *p < 0.05, **p < 0.01 compared with the penicillin-treated group; ^#^*p* < 0.05, ^##^*p* < 0.01 compared with untreated cells; one-way ANOVA. Error bars represent the s.e.m.

We next analyzed whether specific inhibition of the RhoA/ROCK signaling pathway would attenuate penicillin-induced endothelial barrier dysfunction. The data indicated that fasudil, a broadly used inhibitor of ROCK^22^, inhibited penicillin-induced endothelial monolayer hyperpermeability and increased stress fiber formation. Pretreatment with 10 μM fasudil hydrochloride significantly suppressed the penicillin-induced upregulation of ROCK1, p-MYPT1 and p-MLC2 expression in HUVECs (Fig. 4a). Consistently with these results, inhibition of ROCK markedly blocked stress fiber formation in response to penicillin (Fig. 4b–g) and significantly diminished penicillin-induced hyperpermeability of FITC-dextran through the HUVEC monolayer (Fig. 4h).

### RhoA/ROCK Signaling Pathway Plays a Role in Penicillin NAHRs

**Figure 5:**
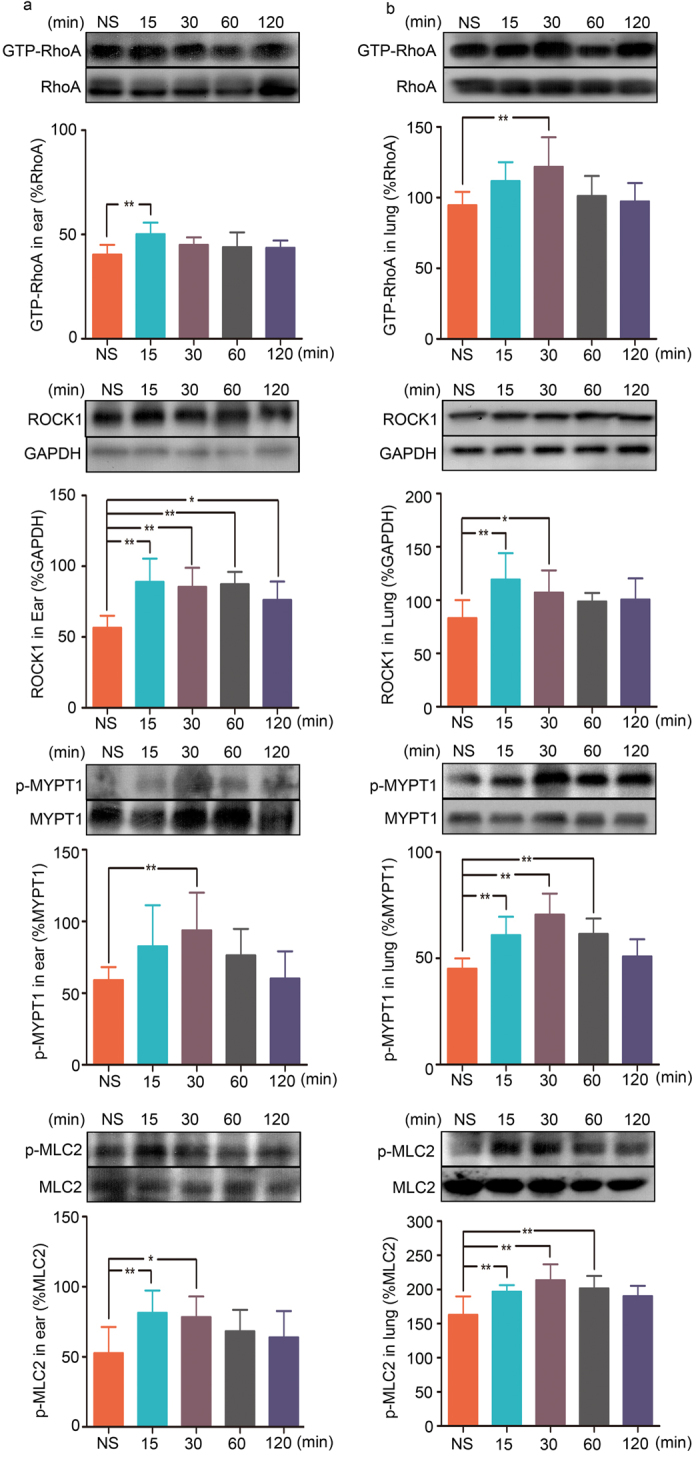
Expression of GTP-RhoA, ROCK1, p-MYPT1 and p-MLC2 in the ears or lungs of mice treated with penicillin. (**a**) GTP-RhoA, ROCK1, p-MYPT1 and p-MLC2 expression in the ears of mice treated with 500 kU/kg penicillin for different time courses (n = 6, per group). (**b**) GTP-RhoA, ROCK1, p-MYPT1 and p-MLC2 expression in the lungs of mice treated with 500 kU/kg penicillin for different time courses (n = 6, per group). NS: normal saline. *p < 0.05, **p < 0.01 compared with the normal saline-treated group; one-way ANOVA. Error bars represent the s.e.m.

Because the RhoA/ROCK signaling pathway is involved in penicillin-induced endothelial hyperpermeability, we next verified the possible contribution of this pathway in penicillin-induced vascular leakage, which is closely associated with penicillin NAHRs. GTP-RhoA, ROCK1, p-MYPT1, and p-MLC2 expression levels were markedly increased in a time-dependent manner in the ears and lungs of mice treated with a single dose of 500 kU/kg penicillin. These proteins were expressed at peak levels 15–30 minutes after penicillin treatment and then gradually declined but did not reach baseline levels until 120 minutes after penicillin injection (Fig. 5a,b).

**Figure 6:**
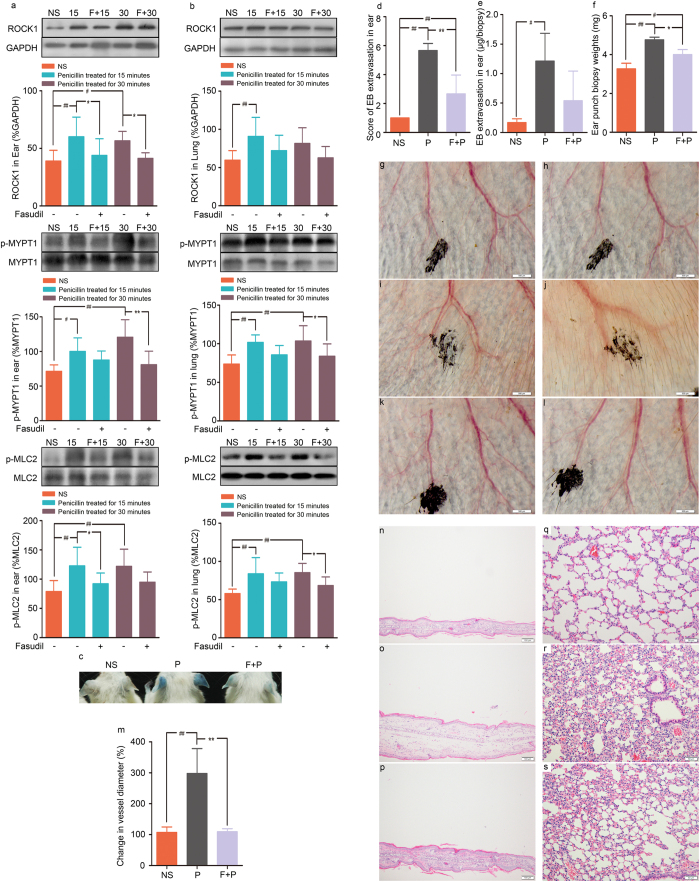
The inhibitory effect of fasudil hydrochloride on penicillin-induced upregulation of ROCK1, p-MYPT1 and p-MLC2 expression in the ears and lungs of mice and NAHRs in mice. (**a**) ROCK1, p-MYPT1 and p-MLC2 expression in the ears of mice treated with 500 kU/kg penicillin with or without pretreatment with 30 mg/kg fasudil hydrochloride (n = 6, per group). (**b**) ROCK1, p-MYPT1 and p-MLC2 expression in the lungs of mice treated with 500 kU/kg penicillin with or without pretreatment with 30 mg/kg fasudil hydrochloride (n = 6, per group). (**c**) Vascular leakage in mice received penicillin/EB with or without pretreatment with fasudil hydrochloride. (**d–f**) EB extravasation for ears and weights of ear punch biopsy for mice received penicillin/EB with or without pretreatment with fasudil hydrochloride (n = 6, per group). (**g–l**) Vascular change of ear ((**g,h**) pre-dose and 30 minutes after normal saline dosing; (**i,j**) pre-dose and 30 minutes after penicillin dosing; (**k,l**) pretreatment with fasudil hydrochloride, and pre-dose and 30 minutes after penicillin dosing). (**m**) Change in vessel diameter (n = 6 per group). (**n–p**) Microscopic examination of ears ((**n**) normal saline; (**o**) 500 kU/kg penicillin; (**p**) 30 mg/kg fasudil hydrochloride +500 kU/kg penicillin). (**q–s**) Microscopic examination of lungs ((**q**) normal saline; (**r**) 500 kU/kg penicillin; (**s**) 30 mg/kg fasudil hydrochloride +500 kU/kg penicillin). NS: normal saline; P: 500 kU/kg penicillin; F + P: 30 mg/kg fasudil hydrochloride +500 kU/kg penicillin. *p < 0.05, **p < 0.01 compared with the penicillin-treated group; ^#^*p* < 0.05, ^##^*p* < 0.01 compared with the normal saline-treated group; one-way ANOVA or Kruskal-Wallis H test. Error bars represent the s.e.m.

We further explored the effect of fasudil on penicillin NAHRs in mice. Pretreatment with fasudil hydrochloride significantly attenuated the penicillin-induced augmentation of ROCK1, p-MYPT1 and p-MLC2 protein levels in the ears and lungs of mice (Fig. 6a,b). Notably, when mice were pretreated with fasudil hydrochloride, the vascular leakage increase, ear swelling, and pulmonary inflammation evoked by a single dose of 500 kU/kg penicillin were markedly alleviated. Penicillin-induced vascular hyperpermeability, increased ear weight, vasodilation and edema were significantly prevented by pretreatment with fasudil hydrochloride (Fig. 6c–m). Moreover, histological observations confirmed that edema and inflammatory exudation in the ears and lungs were largely eliminated in the fasudil hydrochloride pretreatment group (Fig. 6n–s).

## Discussion

Whether penicillin causes NAHRs in addition to immediate AHRs is unknown. In the current study, we demonstrated that the first exposure of mice to penicillin through intravenous injection rapidly provoked significant vascular hyperpermeability. Because typical symptoms of penicillin immediate hypersensitivity reactions, which include rash, urticaria, angioedema, bronchospasm, and anaphylaxis are related to the skin and respiratory systems, we chose the ear (skin) and lung as indicative organs to evaluate the underlying mechanism of penicillin hypersensitivity reactions. As a consequence of penicillin treatment, tissue edema and exudative inflammation appeared in the ear and lung. Prior sensitization with penicillin did not enhance the vascular hyperpermeability reaction. No abnormal signs were observed during the penicillin sensitization phase. There was no evidence of penicillin-specific IgE elicited by penicillin sensitization, as determined by PCA tests. Additionally, penicillin directly stimulated histamine release, and loratadine markedly attenuated penicillin immediate reactions in mice. Thus, penicillin-induced vascular hyperpermeability in mice might be NAHRs. FDA guideline on immunotoxicology evaluations indicates that when signs of anaphylaxis are observed in animal studies, follow-up studies should be performed. However, no effective and validated animal experimental models for NAHRs have been available^33^. This model is the first animal model of penicillin NAHRs, which mimics aspects of the clinical manifestations of penicillin immediate allergic reactions.

As a low molecular weight drug, penicillin is incapable of producing an immunogenic response. Instead, penicillin or metabolites could act as haptens binding to carrier proteins to induce a specific antibody response^34^. Previous animal studies have shown that cutaneous anaphylactic reactions can be provoked by using penicillin/metabolite-protein conjugates in sensitized animals^35^,^36^,^37^. In this study, because we sought to determine whether penicillin induces vascular hyperpermeability independent of IgE, mice were treated with penicillin without protein conjugates. According to the results of the current study, no specific IgE was produced in the mice treated with penicillin that presented induced vascular hyperpermeability.

The dosing instructions for commercially available penicillin injections indicate that penicillin is clinically used at doses varying from 2 × 10^6 ^units/day to 2 × 10^7 ^units/day for intravenous infusion. The doses of penicillin used in mice (125, 250, and 500 kU/kg) in this study were within the clinical dose range according to the conversion of animal doses to human equivalent doses^38^. Apparently, as indicated in this study, penicillin-induced vascular hyperpermeability in mice occurred in a dose-dependent manner. It could be reasonably speculated that some of the penicillin reactions are elicited by high doses.

Agents directly activate mast cells and basophils in an IgE-independent manner, and elicit the release of histamine and other inflammatory mediators, is one of the primary reasons for the occurrence of NAHRs^15^,^17^. Histamine release is reported to be responsible for NAHRs caused by opioid drugs^39^, iodinated contrast material^40^, bradykinin^41^, vancomycin^42^, and polymyxins^43^. In this study, penicillin induced histamine release in both *in vitro* and *in vivo* experiments, and anti-histamine drug diminished penicillin-induced NAHRs. Therefore, our results indicate that penicillin directly elicits histamine release, which may be related to penicillin-induced NAHRs.

Clinical manifestations, including rash, urticaria and angioedema, which occur most frequently in patients with immediate penicillin hypersensitivity, are the consequences of vascular hyperpermeability. Vascular permeability is mainly determined by properties of the vascular wall and hemodynamic factors. Endothelial barrier function is one of the main factors influencing vascular permeability^44^. Endothelial permeability is partly mediated by paracellular mechanisms, which are controlled by the dynamic opening and closing of endothelial cell-cell junctions^21^,^45^. Endothelial junctions have been proposed to bind to cytoskeletal and signaling proteins via associated proteins and to play crucial roles in maintaining vascular integrity and barrier function^45^,^46^. The dissociation of vascular endothelial cell-cell junctions, which is usually caused by actin cytoskeleton reorganization, widens the intercellular space, increases vascular permeability, and facilitates fluid and plasma protein movement^21^,^47^. RhoA, one member of the Rho GTPase family, is a key regulatory molecule for organization of the actin cytoskeleton and integrity of the intercellular junctions^48^. RhoA induces the formation of actin stress fibers at least in part by increasing the phosphorylation of MLC, which is mediated by its downstream effector ROCK^49^,^50^. When RhoA is in an activated GTP-bound form, it binds ROCK, thereby enhancing ROCK’s kinase activity^51^. ROCK directly phosphorylates MLC. In the same time, ROCK also inactivates myosin light chain phosphatase (MLCP) by phosphorylating the myosin-binding subunit (MYPT1), which is considered as a hallmark of ROCK activity^44^,^52^. Both functions of ROCK would result in phosphorylation of MLC, which in turn lead to stress fiber formation^18^. Our results indicate that penicillin promotes actin stress fiber formation and assembly in HUVECs and increases the permeability of the endothelial monolayer. This effect leads to fluid leakage from the circulatory system into the interstitial space, thus resulting in interstitial edema and exudative inflammation, as observed in the ears and lungs of mice treated with a single dose of penicillin. Penicillin-induced exudation in the ears and lungs of mice and HUVEC monolayer hyperpermeability were accompanied by increases in GTP-RhoA, ROCK1, p-MYPT1 and p-MLC2 levels. Pretreatment with the ROCK inhibitor fasudil hydrochloride significantly blocked penicillin-induced endothelial cell stress fiber reorganization and attenuated endothelial permeability in the confluent HUVEC monolayer. Consistently with these results, penicillin-induced vascular hyperpermeability in mice was also alleviated by fasudil hydrochloride. Thus, this study strongly suggests that the RhoA/ROCK signaling pathway plays an important role in penicillin NAHRs. Meanwhile, vascular dilation is also one factor affecting vascular permeability. Vasodilation elevates blood flow and increases the intravascular pressure, which results in hyperpermeability of vascular barrier^53^. It has been reported that the vascular hyperpermeability induced by many inflammatory agents such as histamine^54^ and bradykinin^55^ are related with vasodilation. The present study reveals that penicillin provokes microvascular dilation immediately after the first exposure, which is associated with penicillin-induced vascular leakage and also responsible for penicillin NAHRs.

Approximately 10% of the worldwide population report a history of penicillin allergy, although only a few of these patients have been confirmed to be truly allergic^1^,^2^,^3^. Currently, the widely accepted main reasons for the high misunderstanding of penicillin allergy include the lack of previous clinical diagnoses for patients labeled as allergic, the dependence of the “allergy” symptoms on the illness or interactions between the illness and penicillin, and the attenuation of penicillin-specific IgE over time^3^. However, on the basis of the results of this study, we consider that penicillin causes NAHRs in addition to well-known IgE-mediated AHRs. This underlying mechanism may also contribute to the low reproducibility of penicillin allergies. When patients’ immediate reactions are NAHRs instead of AHRs, the penicillin-specific IgE could not be detected in further diagnosis of penicillin allergy. In addition, this mechanism may explain some cases in which diagnostic tests are negative despite the presence of immediate reactions to penicillin, which are mainly manifested by urticaria, pruritus, and angioedema^56^. According to our research, NAHRs rapidly occur after the first penicillin exposure in a dose-dependent manner, thus underscoring the necessity of optimizing penicillin administration schemes and dosages. When the penicillin dosage is controlled, it is possible that some patients who have previously exhibited penicillin hypersensitivity would be able to tolerate penicillin and safely use it again. We suggest that the mechanisms of penicillin NAHRs include direct histamine release and RhoA/ROCK signaling pathway activation. Thus, administration of anti-histamine agents or inhibitors of the RhoA/ROCK signaling pathway may be useful in preventive approaches against penicillin NAHRs. Furthermore, this animal model of penicillin NAHRs should be a useful tool for further exploring the mechanisms of drug hypersensitivity reactions. This study reveals a novel mechanism of penicillin hypersensitivity reactions and provides a potential path for the clinical use of penicillin in a subgroup of patients with penicillin hypersensitivity.

## Methods

### Ethics Statement

This study was approved by the Research Ethics Committee of the Institute of Chinese Materia Medica, China Academy of Chinese Medical Sciences, Beijing, China. All experiments were carried out in accordance with the ethical guidelines and regulations for the use of laboratory animals and cell lines. All animal-related procedures adhered to the protocol, which was approved by the Institutional Animal Care and Use Committee of the Institute of Chinese Materia Medica, China Academy of Chinese Medical Sciences, Beijing, China.

### Animals

Specific-pathogen-free male ICR mice were received at 8–10 weeks of age and randomized on a body weight-stratified basis.

### Cell Culture

HUVECs were kindly provided by Dr. Song (Academy of Military Medical Sciences, Beijing, China). RBL-2H3 cells were obtained from the cell bank of Shanghai Science Academy. HUVECs and RBL-2H3 cells were respectively cultured in Dulbecco’s modified Eagle medium containing 10% fetal bovine serum (FBS) and minimum essential medium containing 10% FBS at 37 °C with 5% CO_2_.

### Assessment of Vascular Leakage in Mice

In the sensitization experiment, mice were immunized by intraperitoneal injection (ip) of 0.4 mL of a mixture consisting of an equal volume of normal saline/aluminum hydroxide gel (AHG) (Thermo Fisher Scientific, Rockford, IL, USA) or penicillin (Huabei Pharmaceutical, Shijiazhuang, Hebei, CHN)/AHG (2.5, 10, or 40 kU/animal) on days 1, 3, 5 and 19, and challenged by intravenous slow injection (iv) (approximately 2 minutes) of 0.4 mL of a mixture consisting of equal volume of normal saline/EB, or penicillin/EB (1.25, 5, or 20 kU/animal) on day 33. In the unsensitization experiment, the naive mice received (iv) a single dose of normal saline/EB, compound 48/80 (Sigma-Aldrich, Louis, MO, USA)/EB (0.075 mg/animal), or penicillin/EB at the same doses as those of challenge treatment in sensitized mice.

Vascular leakage was assessed by evaluating blue staining in the ear 30 minutes after drug/EB administration. Each ear was given a score of 1 to 6 according to the blue area in the ear, where “1” denoted no visible blue area, and “2 to 6” represented visible blue areas of <12.5%, 12.5–25%, 25–50%, 50–75%, and >75%, respectively. The ears of each mouse were preserved in 2 mL of formamide for EB extraction.

### Passive Cutaneous Anaphylaxis Test

Mice were immunized (ip) by penicillin/AHG or normal saline/AHG on days 1, 3, 5 and 19 as described above or immunized (ip) by 0.4 mL OVA (Sigma-Aldrich, Louis, MO, USA) (0.8 mg/animal) at the same dose schedule as that of the penicillin groups. Immunization sera were collected on day 33. Fifty microliters of serially diluted sera (1, 1/2, 1/4, and 1/8) was intradermally injected into the dorsal skin of depilated recipient mice. After two hours, the recipient mice were challenged by administration (iv) of 0.4 mL of normal saline/EB, OVA/EB (0.4 mg/animal) or penicillin/EB (1.25, 5, or 20 kU/animal). The PCA test results were considered positive when the blue dorsal region had a diameter of 5 mm or more 30 minutes after the challenge injection.

### Evaluation of Ear and Lung Pathological Changes

Mice were treated (iv) with penicillin (125, 250, or 500 kU/kg) or normal saline and euthanized thirty minutes after dosing. Punch biopsies of the left ears (5 mm diameter) and the left lobes of the lungs (wet lung) were weighed immediately, and then the left lobes of the lungs were dried at 80 °C for 24 hours and weighed again (dry lung). The differences in weight between wet and dry lungs were calculated. The right ears and the rest of the lungs were preserved in formalin for histopathological evaluation.

### Vessel *in vivo* Microscopy

Mice were anesthetized and treated (iv) with 500 kU/kg penicillin or normal saline. The ear vessels were monitored at pre-dose, and at 5, 15, and 30 minutes after dosing. The change in vessel diameter (%) was calculated according to equation (1), where D_t_ and D_0_ represent the vessel diameter at t minutes after dosing and at pre-dose, respectively.


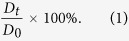


### Histamine Assay

RBL-2H3 cells were stimulated with penicillin (20, 40, or 60 kU/mL) for 30 minutes. The concentrations of penicillin used here were lower than the IC_5_. The supernatants were used as extracellular samples (EC), and the cell lysates prepared by Triton 100-PBS were used as intracellular samples (IC). For histamine assays, 100 μL EC or IC samples were mixed with 50 μL 0.4 mol/L NaOH and 10 μL of 0.1% o-phthalaldehyde methanol solution. After 10 minutes of incubation, 50 μL of 0.5 mol/L HCl was added to stop the reaction^57^. The fluorescence was measured at an excitation wavelength of 360 nm and an emission wavelength of 450 nm using a microplate reader. The ratio of histamine release was calculated according to equation (2) where EC represents EC fluorescence, and IC represents IC fluorescence.


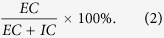


Mice were treated (iv) with 500 kU/kg penicillin or normal saline. Blood was collected 5 minutes after penicillin or normal saline injection and centrifuged to obtain plasma. The histamine concentrations were determined by enzyme-linked immunosorbent assays (Immuno-Biological Laboratories, MN, USA) according to the manufacturer’s instructions. Briefly, the samples were acylated for 1 hour, added to the histamine microtiter plate, and incubated with histamine antiserum for 18 hours at 4 °C. After the plate was washed, it was incubated with enzyme conjugate for 1 hour and reacted with substrate for 20 minutes at room temperature. The reaction was stopped by addition of stop solution to each well. The absorbance was measured using a microplate reader at 450 nm.

### Actin Cytoskeleton Observation

HUVECs were seeded at a cell density of 2 × 10^4 ^cells/well or 1 × 10^5 ^cells/well in a 48-well plate. After cultured for 2 days, cells or confluent monolayer were stimulated with penicillin (1.25, 5, or 20 kU/mL) for 1 hour or pretreated with 10 μM fasudil hydrochloride (Chase Sun Pharmaceutical, Tianjin, CHN) for 30 minutes^58^ and then treated with 20 kU/mL penicillin for 1 hour. The concentrations of penicillin used were lower than the IC_5_. Cells were fixed with formalin for 10 minutes, blocked in normal saline containing 1% BSA for 20 minutes, and stained with 5 μg/mL rhodamine-phalloidin (Sigma-Aldrich, Louis, MO, USA) for 1 hour. Fluorescence images were captured by using a fluorescence microscope.

### Transendothelial Permeability Assay

HUVECs were plated in a transwell insert chambers (0.4 μm pore size, Millicell, Merck Millipore, Cork, IRL) at a density of 4 × 10^4 ^cells/chamber and formed confluent monolayer in 4 days. Then cells were incubated with penicillin (1.25, 5, or 20 kU/mL) for 1 hour or pretreated with 10 μM fasudil hydrochloride for 30 minutes and then treated with 20 kU/mL penicillin for 1 hour. Medium containing FITC-dextran (MW 4 × 10^4 ^kDa, Sigma-Aldrich, Louis, MO, USA) (1 mg/mL) was loaded in the upper compartment of the chamber. FITC-dextran that diffused through the endothelial monolayer to the lower compartment was collected after 10, 20, 30, 40, 50, and 60 minutes^59^. The concentration of FITC-dextran in the lower compartment was measured at an excitation wavelength of 485 nm and an emission wavelength of 510 nm by using a microplate reader. The fluorescence P_app_ (cm/s) was calculated according to equation (3) where [A], t, A, V and [L] represent the cumulative concentration of fluorescence in the receiver compartment (mg/mL), time (s), the area of membrane surface (cm^2^), and the donor concentration of fluorescence (mg/mL), respectively^60^.


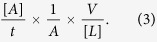


### Effect of Fasudil Hydrochloride and Loratadine on Penicillin NAHRs

Mice were pretreated with 30 mg/kg fasudil hydrochloride (ip) or 3 mg/kg loratadine (Xi’an Janssen Pharmaceutical, Xi’an, Shanxi, CHN) (oral) once daily for three consecutive days. On day 3, mice received (iv) 500 kU/kg penicillin/EB 30 minutes after the last dose of fasudil hydrochloride or loratadine. In the parallel group, mice were treated (iv) with only 500 kU/kg penicillin/EB. Thirty minutes after penicillin/EB injection, the ear scores were evaluated, and the weights of the punch biopsies of the left ears were measured. The punch biopsies of the ears were preserved in 2 mL of formamide for EB extraction, and the right ears and lungs were preserved for histopathology analysis.

In vessel *in vivo* microscopy experiment, mice were pretreated with fasudil hydrochloride (ip) or loratadine (oral) as described above, and treated (iv) with 500 kU/kg penicillin or normal saline. The ear vessels were visualized at pre-dose and 30 minutes after penicillin or normal saline treatment.

### Effect of Penicillin on the RhoA/ROCK Signaling Pathway

HUVECs were plated in a culture dish (10 cm diameter) at a density of 1 × 10^6 ^cells/dish and cultured for 4 days. Cells were incubated with 5 kU/mL penicillin for 5, 15, 30, or 60 minutes, or pretreated with 10 μM fasudil hydrochloride for 30 minutes and then stimulated with 5 kU/mL penicillin for 15 or 30 minutes. Cells were collected and lysed at the assigned time points. Supernatants of lysates were collected after centrifugation at 12,000 × *g* 4 °C for 10 minutes and stored at −80 °C for western blot assays.

Mice were treated (iv) with 500 kU/kg penicillin or pretreated with 30 mg/kg fasudil hydrochloride (ip) and then received (iv) 500 kU/kg penicillin as described above. Ears and lungs were collected at 15, 30, 60, or 120 minutes after dosing in only the penicillin treatment groups or at 15 or 30 minutes after penicillin administration in the fasudil pretreatment groups. Tissue samples were weighed, immersed with 5 times the amount (mL/g) of lysis buffer and homogenized. The supernatants of lysates were acquired as mentioned above.

RhoA activity was assessed by a pull-down assay (Cytoskeleton, Denver, USA) according to the manufacturer’s instructions. Briefly, GTP-RhoA was purified from lysates with the Rho binding domain region of the rhotekin protein bound to glutathione-sepharose beads. The GTP-RhoA was analyzed by western blot assays.

Samples were separated on an SDS-polyacrylamide gel, transferred onto PVDF membranes and probed with primary antibodies of anti-p-MYPT1 (Thr 696) (rabbit polyclonal, 5163, 1:500, Cell Signaling Technology, MA, USA), anti-MYPT1 (rabbit polyclonal, 2634, 1:500, Cell Signaling Technology, MA, USA), anti-p-MLC2 (Thr18/Ser19) (rabbit polyclonal, 3674, 1:500, Cell Signaling Technology, MA, USA), anti-MLC2 (rabbit polyclonal, 3672, 1:500, Cell Signaling Technology, MA, USA), anti-RhoA (67B9) (rabbit monoclonal, 2117, 1:500, Cell Signaling Technology, MA, USA), anti-ROCK1 (rabbit monoclonal, EP786Y, 1:1000, Abcam, Cambridge, UK), or anti-GAPDH (rabbit polyclonal, FL-335, 1:200, Santa Cruz Biotechnology, Santa Cruz, CA, USA) at 4 °C overnight. After the membranes were washed, they were incubated in secondary goat anti-rabbit IgG (H + L) antibody (ZSGB-BIO, Beijing, CHN) at room temperature for 2 hours. Protein bands were detected by enhanced chemiluminescence (Santa Cruz Biotechnology, Santa Cruz, CA, USA). Images of blots were analyzed by ImageJ software.

### Statistical Analyses

The data are expressed as the mean (M) ± standard error of the mean (SEM). All data are independent samples. Statistical evaluations of measurement data (EB extravasation in ear, weights of ear punch biopsy, the differences in weight between wet and dry lungs, change in vessel diameter, histamine release in RBL-2H3 cells, plasma histamine concentration, fluorescence apparent permeability coefficients of FITC-dextran, and protein expression levels) were performed using parametric one-way analysis of variance (ANOVA) followed by LSD (equal variances assumed) or Tamhane’s T2 (equal variances not assumed) multiple comparison test. Ranked data (vascular leakage score) were analyzed with the non-parametric Kruskal-Wallis H test followed by the Mann-Whitney U test. Statistical analysis was performed using SPSS 16.0 software. A *p*-value of <0.05 was considered to be statistically significant.

## Additional Information

**How to cite this article**: Han, J. *et al*. Involvement of Histamine and RhoA/ROCK in Penicillin Immediate Hypersensitivity Reactions. *Sci. Rep.* **6**, 33192; doi: 10.1038/srep33192 (2016).

## Figures and Tables

**Table 1:** Incidence of mice showing positive passive cutaneous anaphylaxis.

Test article	Sensitized dose	Challenge dose	Number of animals (positive response/tested)
Normal Saline	—	—	0/7
Ovalbumin	0.8 mg/animal	0.4 mg/animal	5/5*
Penicillin	2.5 kU/animal	1.25 kU/animal	0/7
Penicillin	10 kU/animal	5 kU/animal	0/7
Penicillin	40 kU/animal	20 kU/animal	0/7

^*^Two animals died during the sensitization phase.
